# A focus on the assessment of the autonomic function using heart rate variability

**DOI:** 10.21542/gcsp.2025.12

**Published:** 2025-02-28

**Authors:** Youssra Amekran, Narjisse Damoun, Abdelkader Jalil El Hangouche

**Affiliations:** Department of Physiology, Faculty of Medicine and Pharmacy of Tangier, Abdelmalek Essaadi University, Tangier, Morocco

## Abstract

Heart rate variability (HRV), the variation in the time interval between successive heartbeats, has emerged as a method to evaluate autonomic function and is increasingly accepted as a biomarker reflecting the balance between the sympathetic and parasympathetic nervous system (SNS and PNS, respectively) branches. Since 1996, most HRV measurements have been performed according to the Task Force standards of the European Society of Cardiology and the North American Society of Pacing and Electrophysiology. However, despite the established guidelines and growing body of research on HRV, this technique has not been fully incorporated into routine clinical practice. This review provides a comprehensive overview of the different aspects of HRV measurement, highlighting the fundamental principles, available methods, and physiological basis of HRV assessment to elucidate its role in understanding autonomic function in normal and abnormal health conditions.

## Introduction

The autonomic nervous system (ANS) is divided into two subsystems: the sympathetic nervous system (SNS) and parasympathetic (PSNS) nervous system. These two ANS branches work together to maintain balance, also called sympathovagal balance, and are responsible for coordinating the body’s unconscious activities as a part of the peripheral nervous system. Among its multiple roles, ANS controls heart rate (HR), blood pressure (BP), respiration, and digestion. ANS activity imbalance is associated with various abnormalities and pathological conditions^1^.

HR variability (HRV), based on the measurement of variation in the time interval between successive R-wave peaks of the QRS complex (R-R intervals), has emerged as a non-invasive and reliable tool for evaluating ANS activity^2^. Research on HRV implications in different clinical and non-clinical settings has significantly increased, especially since the publication of the Task Force report in 1996^3^. It has been associated with morbidity and mortality, stress, and physical performance in many studies^2,3^. Hon and Lee were the first to recognize the clinical potential of HRV when they observed that acute changes in HRV were indicators of fetal distress and predicted foetal hypoxia^4^. At present, monitoring fetal HRV has become a standard of care and has contributed significantly to reducing fetal morbidity and mortality^5^.

The rising interest in HRV can partially be attributed to the feasibility of this method. R-R interval signals can be identified by means of several devices such as electrocardiographs and other sensors, and data are subsequently transmitted and processed by specialized programs developed to process and calculate HRV metrics^6^. HRV metrics are classified as either linear or nonlinear. Linear methods involve time-domain and frequency-domain indices^3,7^. Non-linear indices have been more recently introduced and are suggested to be more reflective of complex biological interactions^8^.

Despite the apparent simplicity and ease of use of HRV, as well as the increasing evidence of the utility of its measurement, challenges and debates remain related to the methodology and physiological inferences that best represent relevant clinical outcomes. With continuing advances in research and technology, our understanding of the origins of HRV has evolved^8^.

This study aims to provide a comprehensive and updated overview of the different aspects of HRV measurement, focusing on the fundamental principles, methods, and physiological basis of HRV assessment to elucidate autonomic function.

## R-R interval time series: The basis of HRV

**Figure 1.**
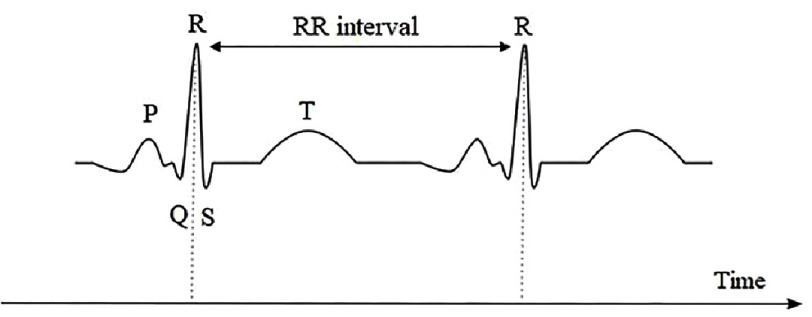
Illustration of the electrocardiogram waveforms and R-R interval.

HR is the number of heart-beats per minute, and HRV consists of the variation in the time between R-R interval series - the time elapsed between two consecutive R waves of the electrocardiogram QRS complex (Figure 1)^9–11^. It has been noticed that the heartbeat frequency is not constant. One may assume that if heartbeats are assessed at a rate of 60 beats per minute (bpm), it should mathematically reflect a rate of one beat per second. However, this is not the case, as the actual rate may vary owing to the difference in time between heartbeats. For instance, the time between heartbeats may vary and may be 0.9 s or 1.2 s, or even more^11^.

It is worth mentioning that HRV analysis does not measure the sinoatrial node’s rhythms, which functions as the pacemaker of the heart, since it is not based on P-P intervals. Instead, HRV reflects fluctuations in atrioventricular conduction superimposed on the P-P interval^12^. Despite this limitation, studies have shown that beat-to-beat changes in the R-R intervals accurately reflect the variability of the sinoatrial node. Ideally, P-P intervals should be used for HRV analysis. However, in practical applications, the small amplitude of the P wave makes accurate determination of the P wave peak difficult, especially in the presence of noise. Consequently, this would impact the accuracy of the P-P interval measurement^12^.

## The duration of recording

HRV can be measured using long-term (≥24 h), short-term (∼5 min), and ultra-short-term (<5 min) recordings^7^. Longer recordings provide a more accurate representation of mechanisms with slower fluctuations, such as circadian rhythms and cardiovascular responses to environmental stimuli. However, short- and ultra-short-term HRV measurements are not directly interchangeable with 24 h values^7^.

Short-term HRV is typically measured in controlled environments, whereas long-term HRV is recorded through Holter monitoring or wearable sensors during daily activities. This distinction is essential for interpreting long-term HRV and its underlying physiological mechanisms^13^.

Frequency-domain methods are commonly used for short-term HRV, while time-domain methods are more suitable for long-term recordings. Nonlinear methods apply to both but have different physiological interpretations, meaning short- and long-term measures should not be considered surrogates^12^.

Long-term HRV is widely used for clinical assessment due to its predictive value^14,15^. However, long-term recordings are influenced by various factors, such as circadian rhythms, the sleep cycle, core body temperature, and the renin-angiotensin system^7^. These recordings have greater predictive power than short-term measurements^7^.

The generation of shorter measurements of HRV stems from the complex interaction between sympathetic and parasympathetic divisions of the autonomic system’ function, respiration-mediated HR increases and decrease through the vagus nerve, referred to as respiratory sinus arrhythmia (RSA), baroreflex, which modulates blood pressure through negative feedback mechanisms, and rhythmic fluctuations in vascular tone^7,14^.

Studies have shown that a decrease in the low frequency to high frequency (LF/HF) ratio in long-term HRV is linked to higher mortality risk^13,16–18^. Additionally, research using HRV and accelerometry data indicates that the LF/HF ratio declines with prolonged periods of lying down over 24 h^13^, suggesting that long-term HRV interpretation must account for activity levels and posture during monitoring. Therefore, evaluating autonomic function through long-term HRV requires simultaneous monitoring of physical activity and posture^13^.

## Ectopic beats, arrhythmias and noise

R-R sequences often contain artifacts, which can vary depending on the recording device and conditions. They tend to be more prevalent during dynamic situations such as exercise^19,20^. HRV measures are highly sensitive to data quality, and artifacts can lead to under- or over-estimations of up to 50%^21,22^.

Artifacts within RR measures can be technical, such as missed beats (inaccurate R-peak detection) and electrical noise (electrode contact issues or movement artifacts), or physiological, such as ectopic beats and arrhythmic events^23^. Ectopic beats are premature beats that produce an abnormally short RR interval, followed by a compensatory delay and prolonged R-R interval^12^. Ectopic beats present a major source of bias in HRV analysis and the interpretation of results^3^. They can compromise the reliability of the HRV power spectrum by producing false frequency components^24^ and affect the accuracy of entropy calculations^25–27^.

To minimize these interferences, pre-processing of HRV data is essential. Common methods include manual artifact correction, which involves visual inspection, signal removal, and interpolation^28–30^. Selecting artifact-free segments or those with an error ratio below 5% is also a common approach^31–33^. Many studies use automated methods followed by manual inspection to verify algorithm accuracy^20^.

Advances in artifact correction techniques have led to various tools and software solutions, but limitations remain. Deletion, linear and cubic spline interpolation, and nonlinear predictive interpolation have been proposed for correcting ectopy. Nonlinear predictive interpolation and deletion generally yield better results than linear or cubic spline interpolation, which can over- or underestimate frequency-domain HRV metrics^25,27^. However, research on the effectiveness of different correction methods remains limited, and many HRV studies do not report the editing methods used.

## HRV analysis methods

HRV measurements can be classified into three categories: time-domain, frequency-domain, and nonlinear (also called fractals or measures of entropy)^3,7^. Time-domain indices, which were among the first to be developed in this field, are still widely used. Frequency-domain indices were later introduced, enabling variability to be differentiated into distinct rhythms based on the frequency. Nonlinear methods are rooted in the premise that HRV is a chaotic time series, and they represent the latest advancements and an active area of research. To emphasize that edited R-R intervals are under analysis, RR intervals are often quoted as N-N intervals, which are normal-to-normal intervals resulting from the removal of ectopic beats and noise from R-R intervals. Both short- and long-term recordings can be analyzed using these methods^34^. A summary of HRV measures using the methods described below is presented in Table 1.

**Table 1:** Summary of the most common HRV indices in time-domain, frequency-domain and non-linear methods.^3,7,23^

Parameter	Unit	Description
**Time domain statistical Methods**		
SDNN	ms	Standard deviation of NN intervals
SDANN	ms	Standard deviation of the average NN intervals for each 5 min segment of a 24 h HRV recording
SDNNI (SDNN Index)	ms	Mean of the standard deviations of all the NN intervals for each 5 min segment of a 24 h HRV recording
RMSSD	ms	Root mean square of successive NN interval differences
NN50	number	The number of NN intervals between which the difference occurs greater than 50 msec throughout the entire record
pNN50	%	Proportion of successive NN interval differences larger than 50 ms
HR Max- HR Min	bpm	Average difference between the highest and lowest heart rates during each respiratory cycle
**Time domain Geometric methods**		
HRV triangular index (HTI)		Integral of the density of the RR interval histogram divided by its height
TINN	ms	Baseline width of the RR interval histogram
**Frequency Domain**		
ULF	ms^2^	Absolute power spectrum of the ultra-low frequency band (≤0.003 Hz)
VLF	ms^2^	Absolute power spectrum of the very low frequency band (0.0033–0.04 Hz)
LF	ms^2^	Absolute power spectrum of the low frequency band (0.04–0.15 Hz)
HF	ms^2^	Absolute power spectrum of the high frequency band (0.15–0.4 Hz)
VLF	%	Relative power spectrum of the very low frequency band (0.0033–0.04 Hz): VLF [%] = VLF [ms^2^] / total power [ms^2^] x 100%
LF	%	Relative power of the low-frequency band (0.04–0.15 Hz): LF [%] = LF [ms^2^] / total power [ms^2^] x 100%
HF	%	Relative power of the high-frequency band (0.15–0.4 Hz): HF [%] = HF [ms^2^] / total power [ms^2^] x 100%
LF/HF		Ratio of LF-to-HF power
LF	nu	Relative power of the low-frequency band (0.04–0.15 Hz) in normal units: LF [nu] = LF [ms^2^] / (total power [ms^2^] –VLF [ms^2^]) x 100%
HF	nu	Relative power of the high-frequency band (0.15–0.4 Hz) in normal units HF [nu] = HF [ms^2^] / (total power [ms^2^] –VLF [ms^2^]) x 100%
Total power	ms^2^	The sum of the energy in the ULF, VLF, LF, and HF ranges
**Non-linear Methods: Poincaré Plot measures**		
SD1	ms	Poincaré plot standard deviation perpendicular to the line of identity
SD2	ms	Poincaré plot standard deviation along the line of identity
SD1/SD2	%	Ratio of SD1 to SD2
S	ms	Area of the ellipse which represents total HRV
**Non-linear Methods: Entropy measures**		
ApEn		Estimation of complexity using approximate entropy which measures the regularity and complexity of a time series
SamEn		Estimation of complexity using sample entropy which measures the regularity and complexity of a time series
**Non-linear Methods: Fractral measures**		
DFA α1		Estimation of signal fluctuations using detrended fluctuation, which describes short-term fluctuations
DFA α2		Estimation of minimum number of variables to define a dynamic model using detrended fluctuation analysis, which describes long-term fluctuations

**Notes.**

bpm beat per minute ms millisecond nu normalized unit

### Time domain HRV

The time-domain parameters of HRV measure the statistical characteristics of R-R intervals or the differences between successive heartbeats, known as delta R-R intervals. These delta R-R intervals represent the sequence of variances between consecutive R-R intervals. Predominantly, time-domain indices rely on statistical analysis of these intervals. In addition, geometric approaches analyse the geometric patterns formed by these intervals^34^.

### Statistical techniques

Statistical measures in the time domain can be derived from a series of recorded R-R intervals. These measures comprise those obtained from direct measurements of R-R intervals or instantaneous HR, as well as those derived from the differences between R-R intervals.

These variables can be obtained from the analysis of the total electrocardiographic recordings or computed using shorter segments of the recording period. The latter technique enables the comparison of HRV during different activities such as rest and sleep^34^.

Statistical time-domain HRV includes the following:

#### The Standard Deviation of NN intervals (SDNN)

The standard deviation of all normal R-R (NN) intervals is measured in milliseconds (ms). ”Normal” indicates that abnormal heartbeats such as ectopic beats, which originate outside the sinoatrial node of the right atrium have been removed^7^. Accurate computation of the SDNN requires meticulous editing to eliminate ectopic beats, artifacts, and missed beats^35^. The SDNN is the most commonly utilized time-domain metric for HRV assessment and is a measure of total variability^3,35^.

In numerous studies, SDNN is computed over a 24-hour period, encompassing both short-term high-frequency fluctuations and the lowest frequency components observed within this period. It is worth noting that as the monitoring period decreases, the SDNN estimates shorter cycle lengths. Additionally, it is important to note that the total variance of HRV enhances with the length of the analysed recording^3,11^.

The SDNN can also be computed from short-term durations, although its reliability is suggested to be higher with long-term recordings. Therefore, comparing SDNN values derived from recordings of varying durations is practically inappropriate. However, to ensure consistency and comparability, it is advisable to standardize the duration of recordings used to calculate SDNN values as well as other HRV measures^34^.

SDNN reflects the combined effects of both SNS and PNS activity. In short-term resting recordings, the main source of variation stems from parasympathetically mediated respiratory sinus arrhythmia (RSA), particularly during slow-paced breathing (PB) protocols (RSA refers to the variability of HR in synchrony with breathing, by which the R-R interval is reduced during inhalation and prolonged during exhalation). In contrast, 24-hour recordings revealed the contribution of SNSs to HRV^36–38^.

#### The Standard Deviation of the average NN intervals (SDANN)

Stemming from the concept of SDNN, SDANN is another statistical variable that captures the variance of the NN. SDANN is calculated over short periods, usually 5 min segments during a 24 h recording, and is measured and reported in milliseconds, similar to SDNN. However, it is important to note that SDANN is not a surrogate for SDNN because it is calculated using 5 min segments rather than the entire 24-h time series^39^. Therefore, it is evident that SDANN is only appropriate for long-term HRV assessments and is usually performed on 24 h recordings^34^. Unlike SDNN, SDANN is less susceptible to editing errors, because averaging several hundred NN intervals reduces the impact of unedited artifacts, missed beats, and ectopic complexity. As such, the SDANN is less affected by the influence of abnormal rhythms^35^.

#### SDNN Index (SDNNI)

SDNNI represents the mean standard deviation of NN intervals within 5-minute segments throughout a 24-hour HRV recording^40^. Thus, this measure estimates the variability influenced by factors affecting HRV within a 5-min period. To compute SDNNI, the 24-h data is divided into 288 segments of 5 min each, with the standard deviation calculated for the NN intervals within each segment, and then averaged across all segments^3,7^. SDNNI is suggested to be a measure of the short-term components modulating HR signals^40^.

While these indices have long been recommended by official guidelines^18^, and have been widely used in studies, new parameters are being incorporated into mathematics and statistics. Among these metrics are those involving normalization techniques, such as the coefficient of variation of NN (CV), that is, the ratio of the standard deviation to the mean, which may provide greater stability when comparing individuals or conditions with varying HR baselines^41^. Additionally, alternative indices are being explored, termed “robust” alternatives to the traditional dispersion indices. Examples of these indices include the Median Absolute Deviation (MAD) or interquartile range (IQR), which relies on the median and is less sensitive to outliers, making them less prone to the effects of measurement errors^40^.

SDNN, SDANN, and SDNNI are calculated directly from the NN series and are considered as deviation-based indices because they are mathematically calculated based on the standard deviation of the NN intervals^40^.

#### Root mean square of successive differences of the NN intervals (RMSSD)

RMSSD is calculated by first determining the difference in time between consecutive heartbeats in milliseconds, then squaring each of these values, and finally averaging the results before obtaining the square root of the total^7^. It is measured in ms^2^. It is also known as SDSD, which represents the standard deviation of successive differences. The measure can be used for long-term recordings, but is more often employed for short-term recordings and is considered the most common time-domain measure of short-term HRV^34^.

RMSSD reflects the beat-to-beat variation in HR and is a time-domain measurement used to evaluate vagally mediated changes in HRV^7,36^. The RMSSD is identical to the nonlinear metric SD1, which is indicative of short-term HRV^42^. RMSSD measurements over 24 h are strongly correlated with pNN50 and HF power^7^. Although RMSSD is correlated with HF power, the influence of respiration rate on this index is uncertain^43^. RMSSD is less influenced by RSA across various tasks^7,44^.

#### The number of interval differences of successive NN intervals that are greater than 50 ms (NN50)

The number of adjacent NN intervals that differ from each other by more than 50 ms (NN50) is a measure of the short-term HRV^7^. This index correlates highly with RMSSD and is an indicator of parasympathetic modulation of the HR^34^.

#### The percentage of successive NN intervals that differ from each other by more than 50 ms (pNN50)

This measure is also used in short-term recordings. pNN50 also reflects the PNS activity. This is correlated with RMSSD and HF power. However, RMSSD provides a better estimate of vagal activity, and most researchers prefer it to pNN50^7,34^.

#### HR Max − HR Min

The average difference between the peak and minimum HRs during each respiratory cycle (HR Max − HR Min) Is particularly sensitive to the effects of respiration rate, independent of vagus nerve activity. To determine the HR Max − HR Min, a sample of at least two minutes is required^7^.

The RMSSD, NN50, pNN50, and HR Max − HR Min are derived from the difference between successive NN intervals; hence, they can be termed difference-based indices^40^.

### Geometric techniques

In addition to statistical methods, geometric methods can also be used to capture time-domain features of HRV, which can be quantified by converting NN interval data into geometric (i.e., graphical) representations. Geometric methods capture information using the probability density distribution of an NN time-series^3,7^.

#### HRV Triangular Index (HTI)

The calculation of the HRV triangular index involves integration of the histogram (i.e., the total number of RR intervals) divided by the height of the histogram, which is determined by the selected bin width. A bin width of 1/128 s has been recommended by the Task Force 1996 to ensure comparable results^3,23,45^.

The HTI is a geometric measure derived from 24 h recordings. A 5-min epoch is conventionally employed to represent this index^46^. The HTI and RMSSD can effectively differentiate between normal heart rhythms and arrhythmias. According to the guidelines, when the HTI is equal to or less than 20.42 and the RMSSD is equal to or less than 0.068, the heart rhythm is considered normal. However, when the HTI is greater than 20.42, the pattern is identified as arrhythmic^46^.

#### Triangular Interpolation of the NN Interval Histogram (TINN)

Another geometric measure is the TINN, which represents the baseline width of the RR histogram determined through triangular interpolation^23^. Similar to SDNN and RMSSD, contamination of a 5-minute segment by only two artifacts can significantly alter its value^47^.

### Frequency-Domain Analysis

**Figure 2.**
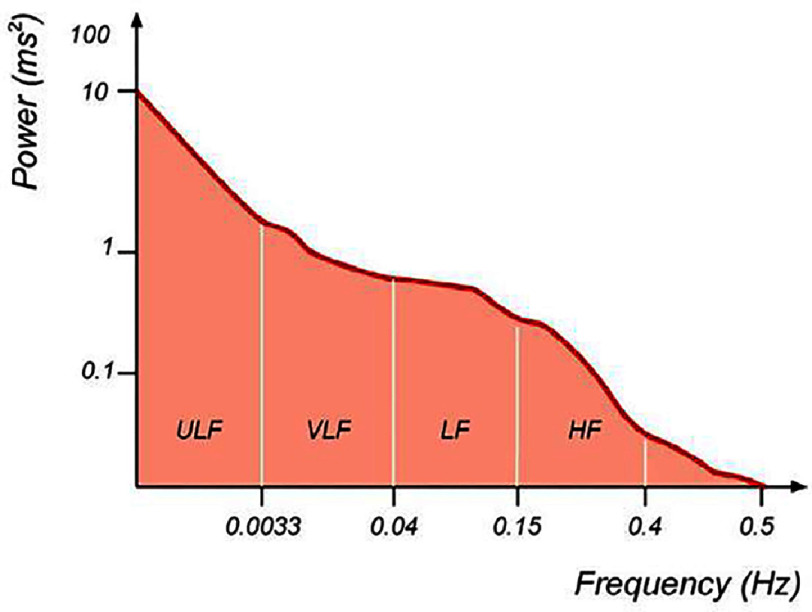
Example of an estimate of power spectral density obtained from a 24 h recording. Adapted from Yılmaz M, et al.,^45^ licensed under CC BY 4.0.

As different regulatory mechanisms modulate HR at different frequencies, frequency domain metrics are particularly advantageous for evaluating the components of HRV. In the frequency-domain analysis of HRV, the focus is on the estimation of the power spectral density (PSD) of HRV as the basis for data extraction. PSD analysis provides insights into the frequency and amplitude of specific rhythms present in HRV waveform^41,48^. This method divides the “power” of a continuous series of beats into its frequency components, identifying them into four bands: ultra-low frequency (ULF; <0.003 Hz), very low frequency (VLF; 0.0033–0.04 Hz), low frequency (LF; 0.04–0.15 Hz), and high frequency (HF; 0.15–0.4 Hz) (Figure 2)^3,49^.

Power spectrum computation can be performed by applying parametric and non-parametric methods. Parametric approaches involve signal modelling using techniques such as the autoregressive (AR) model. Nonparametric methods typically use the fast Fourier transform (FFT) or periodogram for power spectrum estimation^50^.

According to the Task Force^3^, the HRV power spectrum can be divided into four bands: ultra-low frequency (ULF; <0.003 Hz), very low frequency (VLF; 0.0033–0.04 Hz), low frequency (LF; 0.04–0.15 Hz), and high frequency (HF; 0.15–0.4 Hz).

The use of absolute power components, measured in ms^2^, is common in several applications. Additionally, relative power components such as percentages are often utilized. Furthermore, the normalized unit (nu), obtained by multiplying LF or HF by 100 and dividing the product by total power – VLF, can also be employed to measure HF, LF^50^. Other normalization procedures, particularly normalization, are also recommended once the HRV-derived parameters have been obtained. This entails transforming the HRV metrics using a natural logarithm (Ln), which ensures a normal distribution of the parameters^22^.

Different physiological phenomena have been linked to frequency bands. The power distribution over the PSD components is unfixed and changes with autonomic variation of the heart. This is due to the unfixed nature of the PSD function^50^.

#### High frequency power (HF)

The HF (0.15 Hz–0.4 Hz), preferably recorded over a minimum 1 min period, is often known to as the respiratory band, as it corresponds to the HR variations related to the respiratory cycle, commonly referred to RSA^51,52^. The HF component is interpreted as an indicator of vagal modulation and is influenced by the respiratory rate similarities to RSA have also been reported, and is correlated with it (as, *r* = 0.9, *p* < 0.0001)^53^. The relationship between HRV and respiration is well-documented in literature^3,7^. Respiratory parameters (e.g., depth and frequency) are related to HR. Typically, HR accelerates during inspiration and slows down during expiration^54,55^. Two primary physiological mechanisms have been identified in the generation of RSA: modulation of cardiac parasympathetic activity by the central respiratory center and inhibition of vagal efferent activity during inspiration^56^.

The relationship between RSA and vagal tone modulation of HR has generated great interest, and RSA has thus been extensively used as a synonym of the HF band (0.15 to 0.4 Hz, coinciding with respiration rate), which is frequently used as a measure of cardiac vagal tone^55^. Although the neural underpaintings of the relationship between HF and cardiac vagal function^13,57^, there are several factors known to influence the magnitude of RSA independently of vagal cardiac function; thus, questions have been raised as to whether RSA is a genuine index of vagal outflow^13,56^. Respiratory patterns can bias the relationship between the RSA and vagal activity^55^. In general, respiratory frequency is inversely associated with the RSA amplitude (that is, an increase in respiratory frequency decreases the amplitude of RSA). In addition, an increase in tidal volume increases the RSA amplitude^13^. Moreover, RSA measurement can also be affected by physical activity, which can confound the estimation of individual differences in vagal tone. The magnitude of RSA can also be influenced by beta-adrenergic tone^55^. Taylor et al. ^55^ observed that the use of beta-adrenergic blockade increases RSA amplitude across a broad frequency range including frequencies >0.15 Hz, concluding that cardiac sympathetic outflow leads to the reduction of HR oscillations at all frequencies including RSA^58^.

In light of this, it is highly recommended to control or measure respiration parameters to adjust their effects on the measurement of RSA (or HF), and ensure that vagal modulation does not extend beyond the specified HF frequency band^13^. This is particularly important for monitoring populations with slower or faster respiratory frequencies (e.g., athletes, children and adolescents)^13^. Indeed, it has been observed that up to one in five individuals in a sample of healthy participants have been found to breathe at frequencies lower than 0.15 Hz, meaning that including such individuals would bias normal cardiorespiratory conclusions if HF-HRV spectral frequency bands are used^13^. Breathing at a frequency slower than the 0.15 Hz frequency increases the power of the RSA due to baroreflex recruitment. Consequently, this has a significant impact on spectral HRV measures, underscoring the relationship between respiration and HRV computation^13^.

Several investigations have suggested that total vagal blockade eliminates HF power and reduces LF oscillations^51^. Some studies have used pharmacological blockade, such as atropine, and have observed significant reductions in HRV, including the LF and VLF bands, suggesting the contribution of parasympathetic mechanisms (e.g., breathing) to the majority of HRV metrics^51^. However, these observations are challenged by knowing that atropine and similar agent effects extend beyond merely inhibiting parasympathetic activity, but also target the intrinsic cardiac nervous system, particularly the local circuit neurons, involved in cardiac control, and the generation of HRV. Moreover, atropine has been shown to affect sympathetic activity^46^, which suggests that these substances can affect HRV across various frequency bands^51^.

Moreover, proposed solutions, including methods for computing RSA, have been explored, including alternative calculations and statistical correction for varying respiratory patterns. This can be achieved using ANCOVA, with respiratory frequency and tidal volume serving as covariates^56^.

#### Low frequency power (LF)

The LF power range (0.04–0.15 Hz) is typically recorded over a minimum 2 min period^7^. The LF band was previously referred to as the baroreceptor range because it primarily reflects baroreceptor activity under resting conditions^51^. Hence, LF has been used as an index of baroreflex modulation of the heart rate. In addition to the origins of baroreceptor activity, LF was suggested to reflect sympathetic cardiac tone. Evidence relating LF oscillations to sympathetic outflow originates from studies that have explored the impact of orthostatic tilt on HRV indices. Passive tilt is associated with an increase in LF power and decrease in HF power, which suggests an increase in the LF/HF ratio. Additionally, the magnitude of the tilt inclination is highly correlated with both the LF and HF powers^51^. Moreover, a significant association has been established between LF power and sympathetic nerve activity in healthy individuals^13^. However, it is worth noting that this evidence related to the tilt test effect of the LF spectrum concerned only one-third in a trial of healthy subjects, no significant increase in LF was observed in the other third, and a decrease was reported in the remaining third^13^.

Nonetheless, previous assumptions on the origins and clinical significance of the LF component are controversial. Other research hypotheses have suggested that the LF component represents both sympathetic and vagal influences^7,13^.

The SNS does not seem to have a significant influence on rhythms above 0.1 Hz. On the other hand, the parasympathetic system is observed to have an impact on heart rhythms down to 0.05 Hz (20-sec rhythm). Consequently, when the rate of respiration is slow, vagal activity can generate oscillations in heart rhythms that extend into the LF band. These respiratory-related efferent vagally mediated effects are particularly prominent in the LF band when respiration rates fall below 8.5 breaths per minute, which is equivalent to approximately one breath every 7 s, or when an individual takes a deep breath or sight^59^.

Earlier studies have reported that LF oscillations are not completely eliminated by beta-adrenergic blockade, and vagal blockade decreases LF by more than 90%^13^. The LF/HF ratio showed a significant increase with standing; this increase is mainly attributed to the decrease in HF^13^. Additionally, it is important to note that LF power is not correlated with measures of cardiac sympathetic innervation, as assessed by positron emission tomographic neuroimaging^13,60,61^.

Medications that enhance noradrenaline release from the cardiac sympathetic nerves, such as tyramine and yohimbine, have been observed to increase LF power in patients with neuroimaging indications of cardiac sympathetic denervation^62^.

There are different and controversial findings regarding the physiological rationale supporting the assessment of the SNS using LF power. In contrast to the widely held belief that LF power predominantly reflects sympathetic activity, recent studies have demonstrated that it is mediated by both branches of the ANS, in addition to baroreceptor activities.

#### The ratio of LF to HF power (LF/HF ratio)

The LF/HF ratio was originally derived from 24-h recordings. Based on the assumptions that the HF power is commonly used to reflect parasympathetic nerve activity, and that the LF band is often assumed to have a dominant sympathetic component, the LF/HF ratio was proposed to quantify the sympatho-vagal balance. According to this model, which has gained wide acceptance and has been widely used to assess autonomic regulation, an increase in LF/HF power is assumed to correspond to a dominance of sympathetic activity, whereas a decrease in this metric reflects parasympathetic activity dominance^3,7^. Nevertheless, this concept has been challenged as both SNS and PNS activities contribute to LF power^7^. Furthermore, the contribution of the SNS to the LF power is influenced by the testing conditions. Shaffer et al. ^36^ emphasized that various mechanisms can produce 24-hour and 5-minute values of LF power values, which show a poor correlation. For instance, during resting conditions while sitting upright, PNS activity and baroreflex activity are the primary contributors, not SNS activity^63^. Consequently, the interpretation of the 5-min resting baseline LF/HF ratio depended on the specific measurement conditions^7^.

#### Very low frequency power

The VLF (0.0033 Hz and 0.04 Hz) requires a recording period of at least 5 min, but the optimal monitoring spans over 24 h^7^. It is thought to indicate the sympatho-vagal balance; however, the physiological basis and mechanisms underlying the generation of VLF power have not been as clearly delineated as those for the LF and HF components. Despite its predictive power for adverse outcomes, such as cardiovascular disease prognosis, this range of power has been largely overlooked^13,48^.

Long-term regulatory mechanisms, along with ANS activity related to physical activity, the renin-angiotensin system, thermoregulation, and other hormonal factors are suggested to contribute to these VLF oscillations^52,64^. The VLF component elimination was observed with the use of atropine. Consequently, the VLF power is considered an indicator of parasympathetic tone activity^35^.

Furthermore, other findings regarding the origins of VLF oscillations have been reported in the literature. It has been suggested that the VLF rhythm is intrinsically produced by the heart and that the amplitude and frequency of these oscillations are influenced by efferent sympathetic activity^7,36,51^. VLF is correlated with the SDNNI time domain within 24-h periods^7,36^.

#### Ultra-low frequency power (ULF)

The ULF falls below 0.0033 Hz. While there is no consensus regarding the mechanisms generating the ULF band power, the circadian oscillation in HR is implicated and may be the primary driver of the ULF power^51^. Moreover, other slow acting biological processes, including core body temperature modulation, metabolism and the renin-angiotensin system contribute to this power^51^.

A strong correlation between SDNN and ULF, VL, and LF band powers, as well as the total power, has been demonstrated. However, the extent of this correlation varies depending on the measurement conditions. When the ULF, VLF, and LF bands have greater power than the HF band, they contribute more significantly to the SDNN^7^.

### Non-linear methods

Given the variety of factors affecting HR, it is assumed that HR regulation is one of the most complex physiological systems^34^, and the interactions of the mechanisms underlying the cardiovascular system were emphasized to be non-linear in nature^40^. Moreover, HR dynamics are nonstationary, complex, and non-random processes. The stationarity signifies that the statistical properties of the signal remain invariant throughout the recording period^65^. These assumptions motivated the introduction and application of nonlinear techniques to HRV^34^. The nonlinear methods employed include fractal measures (e.g., power Law Exponent, detrended fluctuation analysis), entropy measures (e.g., approximate entropy, sample entropy), and Poincaré plot^34^.

Drawing on the Power Law Exponent index, analysis of the long-term recordings of HRV indicated that 95% of the spectral power is concentrated at frequencies below LF. According to the analysis of these recordings and at these very low frequencies, the spectrum follows power-law behavior. In healthy subjects, the exponent of this power law is close to −1^66^. The power Law Exponent elucidates the nature of the single-frequency characteristics in a time series. When the exponent is equal to 1, it signifies that the time series exhibits similar oscillations acting across varying scales, regardless of the size of the variation (that is, it is “scale invariant”) a property commonly observed in fractals^8^. This concept has previously been employed to analyze the dynamics of beat-to-beat intervals during aging^8,67^.

The approximate Entropy (ApEn) is a measure of the degree of irregularity or randomness within a series of data, yielding an ApEN value ranging between 0 and. Lower ApEn values indicate greater regularity, whereas higher values indicate greater randomness and system complexity^8,34^. In Healthy middle-aged subjects, ApEn values close to or slightly over 1 were observed^34^. This index has been newly applied in biological system signal studies and requires further exploration^8^.

The Detrended Fluctuation Analysis (DFA) method has gained more interest in the literature than other nonlinear techniques. DFA was developed to distinguish between the internal variations generated by complex systems and those caused by external environmental stimuli^65,68^. This is a scaling measure of the correlation between successive RR intervals over a time series^7^. This analysis results in a short short-term scaling exponent, DFA-*α*1, describing brief fluctuations and DFA-*α*2, describing long-term fluctuations^65^. The body of knowledge of the physiological interpretation of DFA-*α* values is not illuminating, although hypotheses suggest that the short-term correlations obtained through DFA reflect the baroreceptor reflex, while long-term correlations reflect the regulatory mechanisms that limit beat cycle fluctuations^7^.

The advantages of DFA over other methods are that it is capable of detecting long-range correlations embedded in time series that may appear non-stationary. Research indicates that DFA may provide more powerful information on the risk of fatal cardiovascular events^16,65^.

**Figure 3.**
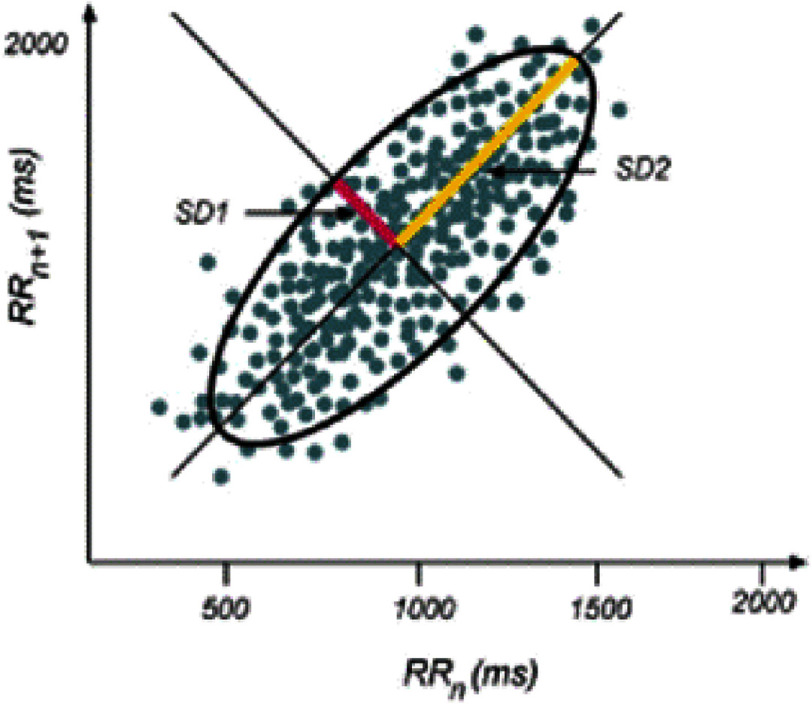
Example of the Poincaré plot map. Consecutive R-R intervals are plotted on the x axis versus next interval R-R n+1 in the y axis. SD1 and SD2 are the dispersion measures along with minor and major axes of the fitted ellipse, respectively. Adapted from Yılmaz M, et al.,^45^ licensed under CC BY 4.0.

The Poincaré plot is a two-dimensional graphical representation of the correlation between successive RR intervals^69,70^. This corresponds to a scatterplot that depicts each NN interval plotted against its corresponding preceding interval (i.e., each interval is plotted on the *x*-axis versus the next break (RRn+1) on the *y*-axis), proving an approximation of the cardiac system’s behaviour^40^. (Figure 3). This statistical method transforms RR intervals into geometric patterns, enabling the analysis of HRV through the graphical properties of the data. The distribution of the points resulting from the analysis is presented as an ellipsoid. Data analysis can be achieved qualitatively by evaluating the figure formed by its attractor, which reflects the degree of complexity of the RR intervals^70–72^. Hence, the presentation of the Poincaré plot provides a visual graphical summary of the heart’s behavior^40,73^. Using this method, and from the plot, it is possible to easily remove premature heartbeats and other technical artifacts, a characteristic that cannot be accomplished with spectral and time-domain analyses^40^.

Scatter plots generated from the RR interval time series provide insights into the corresponding physiological status and reveal valuable information. For example, the Poincaré plot of healthy individuals typically forms an approximate ellipse^74^. Quantitative analysis is believed to provide more objective and precise physiological information. This is realized by adjusting an ellipse to the figure formed by the plot, from which the minor and major axes of the fitted ellipse are extracted and used as HRV indices, in addition to the SD1/SD2 ratio (SD1 represents the standard deviation of Poincaré plot perpendicular to the line-of-identity, while SD2 refers to the standard deviation of the Poincaré plot along the line-of-identity^75,76^. SD1 is a measure of short-term variability in HR and is a marker of parasympathetic modulation^75,76^. Research has shown that tat SD1 and RMSSD are equivalent, and yield identical information^47^. SD2 reflects long-term variability and presents sympathetic and parasympathetic activities, and has been shown to be equivalent to the SDNN index^70,77,78^. SD1/SD2, which represents the ratio of short-to long-term variability in heart rate, provides a measure of sympathovagal balance and is called the Cardiac Sympathetic Index^45,79^. Another parameter is the fitted ellipse area of scatter points (S), used as a marker of dispersion of the points indicating the level of variation of the HRV^45^.

In principle, nonlinear techniques have been shown to be effective in characterizing complex systems and represent a promising tool for analyzing HRV. However, its application to biological and medical data has not been systematically explored, and there are questions surrounding the suitability of these mathematical measures for analyzing biological systems and their sensitivity in detecting nonlinear fluctuations of RR intervals. Additionally, systematic studies on large populations and standards for the use of these methods are lacking. Hence, their significance and physiological interpretations have not yet been fully understood and established^3,8,34^. However, advancements in measurement technologies for assessing HR and HRV may contribute to the further development and application of these methods^3^.

Some studies have aimed to establish reference values for HRV in diverse populations, employing various sample sizes and characteristics, and various measurement methodologies^80–82^. Consequently, the validity and relevance of these values for clinical outcome prediction remains a subject of ongoing discussion^82^. For instance, normal reference values for short-term HRV in healthy adults are currently lower than the Task Force’s standards and exhibit significant inter-individual differences^3,82^ highlighting the need for updated recommendations on measurement considering the potential influencing factors.

Some of the normal values of the frequently used short- and long-term indices of HRV are presented in Table 2.

**Table 2:** Normal values of some frequently used indexes of HRV. Adapted from Yılmaz M et al.,^45^ licensed under CC BY 4.0, and slightly modified.

HRV index (Unit)	Normal range
**Time measurements in 24-hour recordings**	
SDANN (ms)	127 ± 35
RMSSD (ms)	27 ± 12
pNN50 (%)	20 ± 16
HRV Triangular Index	37 ± 15
**Spectral measurements in 5-min recordings**	
Total Power (ms^2^)	3466 ± 1018
VLF (ms^2^)	627 ± 215
LF (ms^2^)	1170 ± 416
HF (ms^2^)	975 ± 203
LF/HF ratio	1.5 ± 2.0

## Heart rate and heart rate variability

HRV estimated from sequences of R-R intervals is negatively associated with HR^3,83,84^. HRV is HR-dependent; different HR may exert different effects on HRV and, to some extent, can determine HRV values^85^. HRV and HR interactions are physiological and mathematical phenomena^86^.

The physiological relationship is determined by ANS activity; that is, the higher the PNS activity, the slower the HR and the higher the HRV. The mathematical dependence of HRV on HR arises from the non-linear (inverse) relationship between RR intervals and HR^86,87^. Consequently, the same changes in HR cause greater fluctuations in RR intervals for a slower average HR than for a faster one. Moreover, the fluctuations of RR intervals for faster HR may not be as high as those for slower HR because the intervals should have become negative^84^, which can mathematically bias the predictive values of HRV metrics and, hence, ANS effect interpretation. Hence, it is important to consider that standard HRV analysis may be subject to mathematical bias, particularly when individuals have varying HR^86^.

Based on these facts, HRV provides information on two components: HR and its variability^86^. It is difficult to determine which of these two factors plays the primary role in the prognostic value of HRV, or in other words, what is the contribution of HR to the predictive power of HRV^86^. This raises the question of whether HRV metrics should be adjusted for average HR.

In this sense, corrections, also called normalization, of HRV with respect to the average RR interval have been suggested and considered nontrivial to explore whether HRV is HR dependent. This normalization can be achieved by dividing the sequence of RR intervals by the corresponding average R-R interval dividing the standard deviation of the RR intervals by the average R-R interval, or dividing the HRV power spectrum by the average RR interval squared^84^.

By employing this correction method, one can differentiate between physiologically and mathematically mediated changes in HRV, especially after interventions that alter the HR. This correction helps understand the true changes in HRV by accounting for the influence of HR fluctuations^84,86^. Such correction is crucial for HRV investigations after interventions altering HR to differentiate between physiological and mathematical changes in HRV; that is, it does not remove physiological differences in HRV with different HR averages; rather, it merely eliminates mathematical bias^86,88^.

Melenovsky et al. demonstrated that after normalization, metoprolol-induced changes in HRV become insignificant, indicating that the observed increase in HRV following beta-blockade is primarily due to changes in HR^88,89^. Additionally, correction methods used to examine the relationship between HRV and maximal oxygen uptake (VO2 max) revealed significant associations. However, these were mainly attributable to the association between HR and VO2 max, meaning that HR is a more reliable indicator of fitness than HRV^90^.

The relationship between HRV and HR has been further investigated, and modification methods have been developed, allowing the adjustment of HRV for the HR effect and removing the mathematical and physiological dependence. This approach involves dividing or multiplying RR intervals (or HRV spectra) by the average RR interval, thereby reducing or increasing HRV’s dependence on HR, respectively.

When division is applied, HRV at lower HRs is attenuated, while HRV at higher HRs is enhanced, weakening the HRV-HR relationship. In contrast, multiplication amplifies this association, strengthening HRV’s dependence on HR compared to standard HRV^86,88^. The higher the power of the average RR, the stronger the effect on the HRV-HR dependence^88^. This method has been tested in post-myocardial infarction patients, showing varying levels of association between HRV and HR classes, which influence the predictive power for various outcomes^86^. Recent studies suggest that removing HRV’s dependence on HR improves its accuracy in predicting noncardiac mortality, whereas enhancing this relationship makes HRV a stronger predictor of cardiovascular mortality^88^.

The relationship between HRV metrics and HR remains an area of ongoing investigation. HRV, which is typically quantified in both the time and frequency domains, reflects the variation in time intervals between consecutive heartbeats. While it is widely acknowledged that HRV is influenced by HR, the extent to which different HRV metrics are dependent on HR and whether different domains (time vs. frequency) exhibit different dependence relationships between HRV and HR remains a subject of investigation. Hence, the intricate relationship between HR and HRV metrics across different domains warrants further exploration to elucidate the underlying physiological mechanisms and enhance the interpretation of HRV in clinical and research settings.

## Practical clinical applications of HRV assessment

The usefulness of HRV as a marker of ANS functions has been proved by its wide range of clinical applications across various fields. In cardiology, reduced HRV is strongly associated with an increased risk of arrhythmias, myocardial infarction, and sudden cardiac death, which has established HRV as a critical tool for risk stratification in patients with cardiovascular diseases^91,92^. HRV has also proven useful in neurology, where it serves as a marker of autonomic dysfunction in neurodegenerative and metabolic disorders. Conditions such as Parkinson’s disease, multiple system atrophy, and diabetic neuropathy are often accompanied by significant alterations in HRV, reflecting impaired autonomic regulation. These changes can aid in early diagnosis and monitoring disease progression^93,94^.

Psychiatric research has also explored HRV as a biomarker for stress, anxiety, and depression, reflecting autonomic imbalance^95^. Moreover, HRV is increasingly used in sports medicine to monitor athlete recovery and training adaptation, guiding individualized training programs to optimize performance and prevent overtraining^96^.

Emerging evidence suggests that HRV could play a role in predicting disease progression and treatment response. For example, in heart failure, monitoring HRV trends may help assess the effectiveness of *β*-blocker therapy, enabling clinicians to adjust treatment plans accordingly^11^. Additionally, in critical care settings, HRV-derived metrics are being investigated for early detection of sepsis and autonomic dysregulation offering a potential tool for improving patient outcomes in intensive care units^97^.

Despite its potential, the clinical implementation of HRV is still hindered by several challenges. Addressing these limitations is essential for the robustness and accuracy of HRV assessments and interpretation.

## HRV measurement challenges and future directions

Despite the extensive use of HRV as a biomarker for ANS activity, several challenges remain regarding its measurement reliability^7,98^. These challenges include the influence of confounding factors, such as age, sex, circadian rhythms, physical activity, and recording conditions, which introduce significant variability and potential interpretation bias^98,99^. Hence, controlling for these variables is complex but essential to ensure accurate and meaningful HRV measurements. Additionally, inconsistencies in data acquisition protocols—such as differences in recording devices, measurement durations, and breathing patterns—further hinder cross-study comparisons. The lack of standardized protocols and guidelines remains a key limitation in HRV assessment^3,7^.

Moreover, nonlinear methods for HRV measurement have gained attention for their ability to capture complex physiological dynamics. However, their physiological significance remains unclear, and the lack of consensus on interpreting these indices limits their clinical and research applications^100,101^.

Furthermore, as the use of animal models is a promising field for understanding a plethora of physiological mechanisms, HRV analysis in mice and other animal models has provided valuable insights into autonomic regulation. However, some limitations such as species-specific differences in autonomic function and HR dynamics pose challenges for the use of animal models for translational research in the field of HRV^102^.

Finally, advancements in artificial intelligence and machine learning techniques offer promising solutions to many of these challenges. AI algorithms, particularly deep learning models like convolutional neural networks (CNNs) and recurrent neural networks (RNNs), can effectively filter noise from HRV signals caused by motion artifacts, poor sensor contact, or environmental factors. This improves the quality of raw data, leading to more reliable HRV metrics^103^. Furthermore, these techniques can automate artifact detection, feature selection, and classification of autonomic states with greater accuracy, enhancing the interpretability and applicability of HRV analysis in both clinical and non-clinical settings^104^.

## Conclusions

HRV is a widely used method for assessing ANS function in both healthy and diseased populations. Its ability to reflect autonomic activity is a growing area of research. HRV measures variations in R-R intervals through time-domain, frequency-domain, and nonlinear methods, each with distinct capabilities in capturing ANS activity.

Time-domain features, particularly SDNN and RMSSD, are the most commonly used, especially in short-term recordings, with RMSSD being the most reliable indicator of vagal tone. However, they are highly sensitive to outliers and artifacts, complicating HRV analysis. Frequency-domain measures provide a more precise quantification of autonomic function, with HF power being strongly linked to vagal tone. However, frequency-domain analysis is affected by methodological concerns, including shorter window inaccuracies and respiratory influences on HF power.

While time- and frequency-domain methods (linear approaches) offer valuable insights, they may be simplistic framework o reflect the complex interplay between sympathetic and parasympathetic activity. Nonlinear methods, developed to better capture these interactions, have shown promise despite limitations, particularly the lack of standardized guidelines for optimal use.

Despite encouraging findings, several challenges remain. HRV metric values vary significantly between studies, underscoring the need for standardized measurement and analysis methods. Additionally, HRV’s dependence on HR is often overlooked, though correction methods have been proposed to adjust for this effect and improve HRV’s predictive value.
